# Women in chemistry: Q&A with Dr Yee Hwee Lim

**DOI:** 10.1038/s42004-024-01320-1

**Published:** 2024-10-28

**Keywords:** Biocatalysis

## Abstract

Dr Yee Hwee Lim currently leads a team of more than 25 researchers in the Chemical Biotechnology and Biocatalysis division at the A*STAR Institute of Sustainability for Chemicals, Energy and Environment (A*STAR ISCE^2^) to develop integrative technologies at the interface between chemistry, biology, informatics, and engineering for sustainable chemical manufacturing.

Dr Yee Hwee Lim has more than 15 years’ experience advancing chemistry frontiers and harnessing nature’s catalytic powers to solve molecular problems. Her research interests include biocatalyst development, chemoenzymatic synthesis and informatics-driven molecular design.

**Figure:**
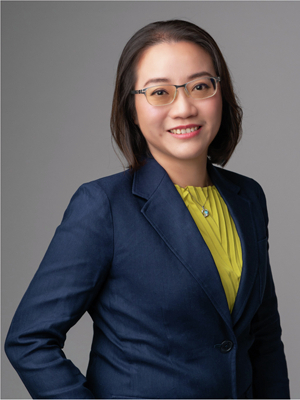
Yee Hwee Lim

Yee Hwee obtained her joint PhD/DPhil from The Scripps Research Institute, USA and the University of Oxford, UK, where she was trained in natural product total synthesis (Prof. KC Nicolaou) and fluorination radiochemistry (Prof V Gouverneur), respectively.

She is also an adjunct Associate Professor at the Synthetic Biology Translational Research Program, Yong Loo Lin School of Medicine, National University of Singapore (NUS) (2021–present). Other previous appointments include Adjunct Assistant Professor at the LKC School of Medicine, NTU (2013–2018) and Director of Graduate Affairs, SERC at A*GA (2020–2023), and editorial member of A*STAR Research magazine (2021–2023).

Why did you choose to be a scientist?

When I was young, I did not want a job that bound me to my desk all the time. So, I chose to be a scientist—a job that allows me to do new things (experiments) every day and is intellectually stimulating.

What scientific development are you currently most excited about?

My team recently delved into the topic of the discovery and engineering of PET plastic depolymerization enzymes. Recently, in collaboration with computational/AI scientists, we discovered an enzyme mutant through a protein language modelling approach that can surpass the state of the art (*unpublished*).

What direction do you think your research field should go in?

My research currently focuses on advancing and applying biocatalysis to make molecules more sustainable. Looking ahead, the speed of development of biocatalysts will be accelerated through integration with automation-driven and AI-enabled approaches. In terms of the next breakthrough in biocatalysis, I see harnessing the best of both chemistry and nature’s catalysis as the exciting future to create new-to-nature enzymes to streamline molecular synthesis.

How would you describe your research philosophy?

My research philosophy is to harness the sparks of interdisciplinary expertise to solve problems. I strongly believe the power of the sum is greater than its parts and a collaborative team with deep individual expertise can do wonders.

What aspects of your research do you find most exciting or most rewarding?

The most exciting aspect of my research is to solve long standing problems or challenges in a field. For example, the halogenase work that we published in 2024 (https://www.nature.com/articles/s42004-023-01083-1) involves an enzyme that was reported more than 20 years ago but was never applied. We realized that it was probably due to the challenge of expressing consistent quality of the halogenase that hindered the progress. It is also very rewarding for me to see next-generation scientists in my team advance and gain achievements.

What do you most (and least) enjoy about being a scientific researcher?

I enjoy the most being surrounded by like-minded peers with complementary skillsets. I least enjoy the administrative duties.

Do you have any advice you would like to share with women starting out in chemical research?

My advice would be to find a supportive employer or supervisor that pushes you so you can grow but also understands the challenges you may face in your life-stage. Also, the world is changing very fast these days, so be flexible and adaptable to learn new skills continuously.

Where do you hope to see women in chemistry in 20 years?

I hope to see more women stay in chemistry research post PhD and more breakthroughs and recognitions of women scientists at the highest level—we are starting to see more representation and I believe this trend will continue upwards.

*This interview was conducted by the editors of Communications Chemistry*.

